# The Week's Work

**Published:** 1910-06-04

**Authors:** 


### The Week's Work.

### THE STATE AND THE HOSPITALS.

The Bill which Senator Robert Owen has introduced into the American Senate, to provide for the establishment of a national department of public health, is one that should have the consideration of every hospital worker, for it deals with a problem that sooner or later will have to be tackled in this country. Our hospital system stands far less in need of revision and reform than does our public health service, but it is unquestionable that the establishment of a Minister of Public Health will be conducive to the best interests of institutions in this country. It is quite true that the functions of such an official, so far as they concern, or have application, to the hospitals and other charitable institutions, are already ably filled by the King's Fund, whose periodical visitations of hospitals have tended so much to keep up a high standard of excellence throughout the English institutional world. From a public health point of view, there is little to complain of in our hospitals, or, at least, in the majority of them. Where in some cases flagrant errors against sanitation, against construction and general principles of hospital hygiene exists—and we have on several occasions had reason in the reports which we have lately published from the pen of one of the best known experts on the subject to point out that some such defects are still to be found—they are due, not to wilful neglect or want of care, but simply to the absence of authoritative opinion which could impress on the committee the urgent necessity of being up to date, thorough, and efficient. So far as the London hospitals are concerned, that authority already exists in the King's Fund. If the suggestion that was dealt with in last week's comments in this department were followed, and every city in the Empire instituted its own local King's Fund, that authority would exist throughout the country, and its value as a stimulus to hospital workers would be inestimable. It is quite possible that a Minister of Health, however energetic, however much respected by all who know his merits, will never be able to influence the institutional world so profoundly as the great funds have done and are still doing, or, to take a closer example, as his late Majesty King Edward was able to do. For all that, the creation of such a special post will be in the interests of the hospitals just as it will be in the interests of the medical profession and the community at large, inasmuch as it will make it possible for concerted action to be taken in regard to many vital questions affecting the health of the community.

### HOW THE HOSPITALS ARE CONCERNED.

With many of these questions the hospitals are closely concerned. Let us for the moment confine ourselves to one point only—the inadequate provision and unsatisfactory regulation of slaughtering animals for human consumption in this country. Every civilised country has already dealt with this matter in a peremptory and scientific manner; we alone have not dealt with it in the only reasonable and proper manner—that is, by placing all slaughter-houses under strict supervision and submitting all animals killed and all meat sold from such places to inspection. Our present system of meat inspection is miserably inadequate, and it cannot be denied that the disadvantages of this system re-act upon the hospitals. He would be a bold physician in a London hospital who dared to give to his patients scraped uncooked meat, such as is frequently prescribed in Germany, where the admirable service of inspection makes it an absolute impossibility for unsound meat to enter a hospital or a private house. Here the hospitals have to see for themselves that the meat which is supplied to them by contract is healthy and sound, and we are convinced, from what we have experienced in hospitals, that it is impossible for them to exercise so rigid and successful a supervision over the quality of the meat supplied to their wards as to obviate all chance of unsound meat being introduced. If a central examining station, which could serve all the hospitals, were desirable in any department of the institutional world, it is surely in this matter of meat inspection, but under ordinary conditions, since the question of the supply of unsound meat does not concern merely the hospitals but the community at large, it is one which the State and not a co-partnership of private charitable associations should undertake. The establishment, therefore, of a Minister of Public Health would enable the Government to attack this problem in a thoroughly workmanlike manner, and through the resulting legislation and supervision, the hospitals, in common with the rest of the community, would inevitably benefit.

### NURSING HOMES.

Our contemporary, *John Bull*, which has always been eager to look into the hospitals, and sometimes imagines that stone walls really make prisons and iron bars cages, even if they are merely ornamental additions to a charitable institution to satisfy the artistic requirements of county and other councils, has found a useful and very efficient safety valve for its tilting zeal in discussing the nursing home question. We wish it every success in its crusade, and willingly place at its disposal whatever thunder we may have evolved in the past in dealing with this important matter. Seriously, the nursing home question is in many respects a scandal, and if a lay paper can arouse the public to the real danger in carelessly allowing many of these so-called homes to flourish without check or inspection of any kind it will have done praiseworthy work for which we will be duly grateful. Our own efforts to touch this subject, limited as they must be to appeals to readers who know the facts as well as we do, but who, with the customary apathy of busy medical men, shrug their shoulders and declare that they cannot see any way out of the difficulty, are far less likely to impress the great outside public than the sensational manner in which our contemporary proposes to deal with the problem. We do not share the usual prejudice against "sensational methods" of appealing to the public or of arousing practical interest in matters of vital importance. Peter Hermit was sensational—not that we wish to compare for a moment the morality of a crusade against the Saracen with that of a crusade against the modern nursing "home"—and was there anything more "sensational" than John Howard's speeches and leaflets or than the *Lancet's* report of the notorious Hounslow flogging case? If our contemporary succeeds in waking the public conscience in this matter of nursing homes no one, we feel sure, will quarrel with its sensationalism.

### THE RITTERSVILLE ASYLUM.

An extraordinary state of affairs is revealed by the newspaper accounts of the procrastination and red-tape that have been responsible for the delay in building the State asylum for the insane at Rittersville, in Pennsylvania. It appears that nine years of time and no less a sum than £550,000 have been expended upon this hospital, and that even as yet not a single ward is fit for occupation. It would be interesting to hear what are the facts about this unheard-of extravagance and inefficiency, which is unparalleled in the history of hospitals, though there is a legend that Bishop Basileus' Hospital for Plague Patients took 19 years to build, and was then only fit for an arsenal! That, however, is a legend, and, although in a sense traditional, it can scarcely be looked upon as authentic history to which hospital chroniclers can attach much credence. "The state of affairs at Rittersville," remarks our contemporary, the *International Hospital Record*, "would be surprising in any State except Pennsylvania, in which politics seem to have reached the acme of rottenness. It is a blessed good thing that Ben Franklin and William Penn are dead!" Those who are enamoured of State-supported hospitals would do well to devote some attention to the conditions which have enabled the authorities to make so brilliant a fiasco at Rittersville.

### THE LATE DR. LENHARTZ.

The death of Professor Dr. Hermann Lenhartz removes from the ranks of German hospital workers one of the best-known men, whose reputation was not merely local or national. Lenhartz was a medical man, specially interested in clinical and therapeutical work, and the first 12 years of his professional life were passed as assistant to more or less forgotten masters of German medicine. His opportunity came to him when he was selected as chief of the then little known University Polyclinic at Leipzig, after several better-known teachers of medicine had declined the call to that post. The Leipzig Medical School is now one of the most flourishing in Germany, and it owes much of its present popularity to the energy and zeal with which Lenhartz tackled its problems. While working there he became interested in hospital construction, and began to contribute articles on the subject to various professional papers. These, it must be confessed, are much less well known than his papers on practical and theoretical medicine. In 1895 he was called to Hamburg, and from that time started his partnership with Herr Ruppel, the best-known German hospital architect. Together they designed the magnificent Hamburg Eppendorf institution, which was, and in some respects still is, an original type, which no one interested in hospital construction can afford to neglect; and later on they planned, on equally successful lines, which had been modified through the invaluable experience gained at Eppendorf, the reconstruction of Lenhartz's old hospital, the St. George's Infirmary. His association with Herr Ruppel reminded one of the intimate association that at one time prevailed between the medical and surgical clinics at Breslau when Naunyn and Mickulicz were at their heads, and certainly it has been as successful from the point of view of hospital construction and organisation as that brilliant co-partnership. Lenhartz's energy was enormous. He worked from early in the morning until late at night, and his knowledge of hospital organisation, management, history, and work generally was astonishingly encyclopædic, while at the same time it was thoroughly practical. With all his work as superintendent and organiser of what was practically a new system of hospital management he yet found time to produce a large amount of original and highly interesting work in clinical medicine, and his pioneering researches on the subject of gastric ulcer are well known to English medical men, though it is to be regretted that his equally useful observations on gangrene of the lung and appendicitis are not so generally recognised by the profession. Personally he was an interesting man to meet, always courteous and kindly to visitors who came to inspect his clinic, and specially so to those who ventured to take an interest in the construction of his hospitals. He was broad-minded enough to recognise that his constructive work was not ideal, and he welcomed criticism. He had a good acquaintance with English hospital literature, although he confessed that he had never had time enough to visit the best English institutions, though we believe that he made a tour of inspection in London and the provinces shortly after he took over the work at Eppendorf.

### THE DRUG MARKET.

A quiet condition still prevails in the drug market, although there is some sign of pending improvement. A drug which is being watched with interest at the moment is opium, as it is anticipated that the forthcoming Turkish crop will be large; if this proves to be correct, lower prices may be expected, in fact the decline in value may be substantial. Naturally buyers will refrain from making purchases until more definite information is to hand. Quicksilver has again been reduced in price, and makers of mercurial salts have in consequence reduced their quotations, corrosive sublimate, calomel, white precipitate, red precipitate, and yellow oxide of mercury being one penny per lb. cheaper. Thymol is about 1s. per lb. lower. Jalap is still tending dearer. Cod-liver oil still exhibits a tendency to decline in value. Orris root is dearer. Camphor is without change in price, but it is probable that any alteration would be in an upward direction.

